# Editorial: What is addictive in gambling? Nine years after the gambling disorder diagnosis: Does gambling fit among the addictions?

**DOI:** 10.3389/fpsyt.2023.1147345

**Published:** 2023-02-15

**Authors:** Anders Nilsson, Jakob Jonsson

**Affiliations:** Department of Clinical Neuroscience, Karolinska Institutet (KI), Solna, Sweden

**Keywords:** gambling, addiction, behavioral addiction, DSM-5, Gambling Disorder (GD)

With the transfer of the Gambling Disorder (GD) diagnose from an impulse control disorder into the *addictive disorders* chapter of DSM-5 (1), it wasn't so much of a watershed moment as a confirmation of what researchers, patients and clinicians had asserted for decades: gambling can be an addiction just as any drug or alcoholic beverage. Nevertheless, the transfer likely had an important impact on public awareness and, to some extent, government policy. It also sparked a more intense discussion on how we define addiction (2). In its most simplistic conceptualization, addictions stem from the addictive properties of certain substances' molecular structure. While most researchers understand and recognize the broader and more complex mechanisms at work, the substance inhaled, injected or ingested is generally understood as the main driver of addiction. GD challenges this assumption, and its reconceptualization as an addiction introduced the theoretical possibility for virtually any behavior to be termed as potentially addictive.

However, there are still many fundamental aspects of GD, its prevention and its treatment, that remain understudied compared to substance use disorders (SUDs). One such area is pharmacological treatments, of which none has FDA approval, for instance. However, as is described in the article by Tjernstöm and Roman, in gambling tasks for male rats, gambling and alcohol intake, but not elevated sexual behavior, seem to share underlying mechanisms among. It also showed that naltrexone lowered the rats' propensity to “gamble”, which shows that naltrexone holds some promise as treatment, even though there is a long way to go from rats to humans.

Another example of how gambling treatment could be inspired by the treatment of SUDs is the use of quality registers. As described by Håkansson and Åkesson, unlike treatment for SUD, interventions for GD are not always fully developed or integrated into health care services. The authors' introduction of a quality register for GD into the Swedish health care system in order to better monitor treatment uptake and needs could provide us with a better understanding on how to reach target groups and tailor our interventions. The issue of low treatment seeking is a longstanding issue within the GD research field, but several studies indicate that a large proportion of individuals with problematic gambling behaviors attempt to change their gambling on their own. In a study on more than 10,000 people, Hodgins et al., found that 90% of individuals with GD indicated that they tried to change primarily on their own. Around one third indicated that they feel too ashamed to seek help. This could partly be related to the fact that many individuals with GD gamble to escape everyday problems, as shown in the study by Neophytou et al.. Raising awareness about GD and offering accessible and reliable treatments for GD is essential to reduce shame and lower barriers to treatment, but it is obvious that many would still prefer to handle problems on their own.

The gambling research field is increasingly using industry data to analyze gambling patterns, risk factors and prevention tools offered with in the games. Pop-up messages, as described by Caillon et al., could be part of a wide range of preventive measures to prevent problem gambling at an earlier stage, especially if implemented with careful consideration and together with other responsible gambling tools. Engebø et al. investigated the use of several such responsible gambling tools (e.g., temporary breaks, taking self-test, etc.) among almost 6,000 gamblers. The results indicated that the tools were used only by a minority of gamblers, but that problem gamblers were more likely to use them than other gamblers. This is an area where gambling research has no blueprint from the SUD research field to follow. While online gambling could increase risks, given its high availability, it could also provide researchers with a unique insight into the mechanisms causing and maintaining GD. For the next 10 years, gambling as well as gambling research, will increasingly be conducted in the online world.

## Author contributions

AN and JJ collectively wrote this editorial. All authors contributed to the article and approved the submitted version.
